# Machine learning approaches in non-contact autofluorescence spectrum classification

**DOI:** 10.1371/journal.pdig.0000602

**Published:** 2024-10-09

**Authors:** Ashutosh P. Raman, Tanner J. Zachem, Sarah Plumlee, Christine Park, William Eward, Patrick J. Codd, Weston Ross

**Affiliations:** 1 Department of Biomedical Engineering, Duke University, Durham, North Carolina, United States of America; 2 Department of Mechanical Engineering and Materials Science, Duke University, Durham, North Carolina, United States of America; 3 Department of Neurosurgery, Duke University School of Medicine, Durham, North Carolina, United States of America; 4 Department of Orthopaedic Surgery, Duke University School of Medicine, Durham, North Carolina, United States of America; Dalhousie University Faculty of Computer Science, CANADA

## Abstract

Manual surgical resection of soft tissue sarcoma tissue can involve many challenges, including the critical need for precise determination of tumor boundary with normal tissue and limitations of current surgical instrumentation, in addition to standard risks of infection or tissue healing difficulty. Substantial research has been conducted in the biomedical sensing landscape for development of non-human contact sensing devices. One such point-of-care platform, previously devised by our group, utilizes autofluorescence-based spectroscopic signatures to highlight important physiological differences in tumorous and healthy tissue. The following study builds on this work, implementing classification algorithms, including Artificial Neural Network, Support Vector Machine, Logistic Regression, and K-Nearest Neighbors, to diagnose freshly resected murine tissue as sarcoma or healthy. Classification accuracies of over 93% are achieved with Logistic Regression, and Area Under the Curve scores over 94% are achieved with Support Vector Machines, delineating a clear way to automate photonic diagnosis of ambiguous tissue in assistance of surgeons. These interpretable algorithms can also be linked to important physiological diagnostic indicators, unlike the black-box ANN architecture. This is the first known study to use machine learning to interpret data from a non-contact autofluorescence sensing device on sarcoma tissue, and has direct applications in rapid intraoperative sensing.

## Author summary

Surgical removal of solid malignant tumor tissue in soft tissue is complicated by the need to remove as much cancerous tissue as possible without also encroaching on necessary healthy tissue. Instead of using external fluorescent dyes for delineation of malignant tissue, newer sensing instruments in the neurosurgical landscape take advantage of internal metabolic processes to accurately and non-invasively outline malignancies throughout the course of a surgery. One portable device, developed by our lab, highlights spectroscopic signatures naturally emitted by cancerous tissue in contrast to healthy nervous tissue, using a purely optical laser source. In this study, we employ and compare various machine learning models to automatically differentiate between device-generated spectroscopic signatures of healthy soft tissue and cancerous soft tissue sarcoma from mice. We achieve highly accurate results using different model architectures, and further, link the results back to actual metabolic processes taking place in diseased tissue in an interpretable manner, allowing for potential application in future rapid intraoperative sensing.

## 1. Introduction

### 1.1 Standard of intraoperative imaging

In precision oncologic surgeries, it is important to be able to continuously identify tumor margins and classify tissue during the course of a surgical resection. This includes the ability in real-time to make this determination even as the anatomic landscape changes as tissue is removed or additional anatomic regions are exposed. Sarcoma, a connective tissue cancer, is one of a variety of malignant tumors that require precise resection to maximize survival and reduce disease progression while salvaging healthy surrounding tissue to minimize damage to important neurovascular and biomechanically important tissues [1–3].

Precise evaluation of this tumor vs. normal classification of tissue can become quite difficult as a procedure progresses, since the surgical manipulation of tissues creates tissue deformation that can make visual distinction between normal and healthy tissues unclear. These tissue deformations can cause deviations from initial preoperative diagnostic imaging of up to 1.6 mm in the vicinity of a lesion, which can directly affect post-surgical outcomes [4–6]. Magnetic resonance imaging (MRI) and computed tomography (CT) remain the main modalities for pre-operative lesion segmentation, but scans created using these modalities become less valuable as surgical regions of interest (ROIs) change due to drug effects and mechanical manipulation through the course of the operation [7]. This leads to a dearth of real-time information during the surgery by which to guide the identification of pathologic and normal tissues. For this reason, there has been much work conducted in intraoperative imaging, to provide updated images of the ROI at different stages of the procedure. In particular, iMRI has become the preeminent method for intraoperative imaging, particularly in neurosurgery.

Although strides are being made in optimizing iMRI acquisition time and portability, it remains quite intensive and prohibitive for surgeons, especially during time-intensive procedures [8]. Surgeries must be halted while iMRI devices are brought in and set up around the patient, or the patient must be transported to an available MRI outside of the operating theater. Additionally, iMRI is an expensive technique, and not suitable for low-resource situations [9]. There remains a need for varied methods of intraoperative tissue diagnosis; these diverse methods attempt to improve upon the problematic acquisition and portability issues of iMRI, while also providing added benefits of near real time acquisition.

### 1.2 Previous use of fluorescence for surgery

One such method for improved intraoperative diagnosis is fluorescence spectroscopy [10–15]. Using fluorescently delineated biological features in pathological and healthy tissue to characterize differences and identify tissue boundaries allows for the capability of machine-based precision that is unachievable with simple visual inspection by a surgeon or radiologist. These techniques allow *in vivo*, real time identification of tumor tissue, where previously, time-intensive intraoperative histopathologic identification would need to be carried out by a pathologist on sampled areas of tissue throughout the case.

Exogenous fluorescence, through means of external, synthetic fluorophores that selectively bind and fluoresce certain sites within cells and tissue, are popular choices for fluorescence guided surgery. For instance, 5-aminolevulinic acid (5-ALA) is an oft-utilized fluorophore in neurosurgery for glioma tumor identification and resection [16–19] while other molecules like tetracycline and cathepsin-activatable Cy5 fluorescent imaging probe (LUM015) have been explored for usage in soft-tissue sarcoma or osteosarcoma fluorescence [20–23]. 5-ALA is an exogenous agent that is preferentially taken up by cancerous glioma cells and converted to protoporphyrin IX (PpIX), a fluorogenic metabolite, through internal cellular processes. While 5-ALA and other external fluorophores can highlight a tumor area under low-power blue light, administration of these contrast agents often coincides with concurrent usage of resource-intensive iMRI, which does not solve the issue of portability and real-time intraoperative diagnosis [16]. Further, use of 5-ALA and similar tumor contrast enhancement agents can adversely affect patient outcomes through systemic effects unrelated to a surgical procedure [24].

Endogenous fluorescence on the other hand, which leverages nascent physiological differences in tissue to diagnose or delineate margins, poses a potential solution to the challenges created by preoperatively- and exogenously-administered promolecules and fluorophores. In the case of malignancies, there are metabolic differences between tumor and healthy tissues that create natural spectral differences that open the way for unique classification. Because malignancies consume sugars at a higher rate than regular surrounding tissue, they create relative differences in important electron carriers, like reduced β-Nicotinamide adenine dinucleotide (NADH) and Flavin adenine dinucleotide (FAD), and other important byproducts of cellular respiration, like lactic acid and porphyrins, a precursor to hemoglobin [25–26]. This finding, known as the Warburg effect, can be highlighted through use of fluorescence spectroscopy. Electron carriers release photons as energy, creating fluorescent emission, when excited by external, near-ultraviolet laser sources. Endogenous fluorescence is being increasingly explored for its ability as a non-destructive, point-of-care tissue evaluation method to aid physician and surgeon insight [12,27]. Selectively-activated near-infrared (NIR) fluorescence has been utilized in other types of solid tumor surgery, most notably soft tissue sarcoma, where achieving a margin-negative resection is critically important [28]. However, near-ultraviolet endogenous fluorescence, which can elicit emission from important carriers like NADH, FAD, and porphyrins without inducing free radical propagation, has not yet been evaluated on sarcoma tissue [29].

**Table 1:** Emission wavelength and full width at half maximum (FWHM) values for various metabolically important endogenous fluorophores after 405 nm laser excitation [30–31].

Fluorophore	Emission Peak (nm)	FWHM (nm)
Free NADH	487	84
Protein-bound NADH	501	64
Free FAD	544	75
Basic form of porphyrins	590	25
Neutral form of porphyrins	630	25

Important fluorophores excited by a 405 nm coherent laser pulse are shown in Table 1, collected in previous studies of autofluorescence for biomarker detection. Emission peak and Full Width at Half Maximum (FWHM) are also provided in Table 1. As shown, previously determined important biomarkers exhibit varying emission peaks depending on factors like pH and chemical binding to other compounds.

### 1.3 Previous fluorescence spectroscopy devices

While autofluorescence is an improvement on previous methods of intraoperative tissue characterization, it still lacks some of the qualities necessary for effective incorporation into the surgical pipeline. Liu et al. describe a device capable of conducting spectroscopy on endogenous fluorophores [32]. While this bifurcated fiber-based non-epifluorescent device corroborates the theoretical use of the Warburg effect to guide esophageal tumor identification, it still involves direct contact with the tissue in question in order to accurately determine if the tissue is neoplastic or healthy. To provide noncontact based advantages like increased acquisition speed, negligible tissue deformation, and wide-field imaging to patients from a reusable device, other methods of detection are necessary [33]. To date, certain devices have begun to be included in clinical workflows through pairing with GI, brain, and skin applications, showing the increasing desire for photonic noncontact sensing [25–26]. However, there is a dearth of noncontact near-UV photonic sensing devices for cancers like sarcoma and other connective tissue cancers, where such capability could be vital for safer and more precise surgical action.

### 1.4 Warburg effect and our previously-created device

To combat the need for direct contact-based fluorescence spectroscopy, Tucker et al. recently developed a system capable of noncontact-based excitation of endogenous fluorophores [34]. This device uses a standard epifluorescence design with a 180mW 405 nm laser to excite the tissue, and the emission is recorded using a CCD spectrometer. The laser spot is focused to a 1/e^2^ spot size of .75mm at a working distance of 17mm. Collected spectral emission data can then be analyzed to identify differences in spectra and create a diagnosis for use by the surgeon. In order for this to be effective, however, the post-processing and classification of tissue must be relatively accurate and quick, so as not to interrupt the surgeon workflow, and in order to provide real-time feedback results akin to those of a pathologist without the necessity of ambiguous tissue resection.

Through the combination of continuous data-streaming from instrument based sensing and rapid information post-processing, this device can provide an accurate and supplemental aid to iMRI or visual inspection by surgeons. However, to date, this device has not been evaluated in its ability for post-processing. There exists a need for machine learning-based classification techniques in order to decide if further device optimization is required, or if this device is even a viable option for tissue classification automation during a surgery.

This device was previously demonstrated to work in automated robotic surgery settings by our group for glioma-mimicking phantoms [34–35]. The device was set on tumor boundaries and allowed to collect spectral signatures, which informed a CO2 cutting laser scalpel on where to preferentially ablate tissue. Further, the precision and effectiveness of the device at the tumor-healthy brain boundary was previously demonstrated through a computational algorithm which takes into account various device-specific parameters, raster patterns, tissue chemistry and tumor shapes [36]. Though these experimental findings were crucial toward initial device development and optimization, the device has since been enhanced and validated through automated machine learning classification and testing on sarcoma tissue, as shown in this study. This study serves as an analysis of the device’s non-contact point-of-care autofluorescence capabilities on biological tissue, as well as its ability to create discernable spectral classes that can be sufficiently classified.

### 1.5 Machine learning and important models

Machine learning poses a helpful combination technique with devices like these, in order to interpret and accurately classify disparate classes of tissue in an automated fashion. Supervised learning, in particular, can be easily used, since ease of validation is high and accuracy, specificity and other metrics can quickly be determined. Trained machine learning models can be stored in a device CPU and then coupled to one such as that of Tucker et al. These devices can then be deployed on unlabeled data, like visually indiscernible tissue in an operating room.

Regardless, it is integral to determine which model classes and specific data pre-processing techniques should be used in order to create optimal prediction capabilities. It is important to compare methods and pipelines in order to determine how simple neoplastic tissue is to classify, and how feasible it is for deployment in an operating room. In order to investigate this question, a number of machine learning methods and pipelines are investigated in this paper, and several evaluation metrics are used to compare methods. Data cleaning is further detailed in Methods. Feature engineering is performed through Principal Component Analysis to reduce dimensionality and hopefully diminish inherent noise in the dataset. Finally, classification techniques include the Artificial Neural Network, Support Vector Machine, Logistic Regression, and K-Nearest Neighbors.

### 1.6 Feature engineering and general model pipeline

Principal Component Analysis (PCA) is a feature extraction technique used to determine and highlight important features within a piece of data. It is effective in dimensionality reduction, especially in sparse or small datasets. In classification techniques, it is paramount to have more data than features per datapoint as this prevents the potential for overfitting of data and poor resulting performance in future algorithmic classification. PCA works by creating a linear transformation of high-dimensional data to a lower-dimensional space through a series of representative basis vectors. This is done in a simple matrix operation akin to Ax = B, where x is the conversion matrix. PCA attempts to identify the main dimensions of variance for a multidimensional dataset, which allows for further data exploration into the differences between data and the identification of important features for a particular dataset.

For classification methods, K-Nearest Neighbors (KNN) provides perhaps the simplest approach, involving use of neighboring data points to classify subsequent ones. It uses proximity in distance and labels of known data to draw a separating boundary between pre-specified classes. One can place unlabeled datapoints in this decision space and make inferences about class affiliation from this. It is used in this study as a baseline model, upon which classification improvements will be evaluated in other models.

Logistic Regression (LR) is also another relatively simple classification model, utilizing a sigmoid function to floor or ceiling probability values into one or more classes. A logits function converts assigned beta coefficients- which are found through a linear combination of predictor variables in a dataset- to probabilities. A threshold probability, usually of .5, is used to determine classification in a certain group, and thus creates discretized outcomes.

Support Vector Machines (SVM) represent a relative increase in complexity of machine learning classification model, as support vectors, or ambiguous points of classification, are utilized to deduce areas in which to craft an optimal decision boundary. SVM occasionally confers better classification abilities than its architecturally simpler counterparts like LR and KNN due to its many hyperparameters that can be tuned to the specific requirements of a dataset. The Radial Basis Function is a nonlinear initialized kernel used by SVM for the best model performance in classification tasks like these. SVM is especially fitting for highly accurate classification, as it allows for decision boundary hyperplane building in the case of otherwise linearly-inseparable data by projecting data to high-dimensional feature spaces. SVM has been used in the past for classification of fluorescence spectroscopic signatures for prostate cancer cell lines [37].

Lastly, the Artificial Neural network (ANN), which we use in our study, is increasingly used in combination with instrument based sensing for its ability to feature engineer, hyperparameter tune, and classify without much user assistance [38–39]. This class of algorithms utilizes multiple epochs with set batches to continuously update a model’s weights, while using the same data in every epoch. Unfortunately, this architecture type prevents studying of the important components within a dataset that contribute to the differentiation of classes. For example, it may be difficult to determine important wavelength-intensity pairs that differ in pathological and healthy tissue, since weights within an ANN do not necessarily correlate to data in an interpretable manner. While these models can be useful, they also require long training times, especially with larger datasets, since decision functions must update constantly and follow an optimization process through gradient descent. Nonlinearities between layers of a network, and cutting of neurons to increase robustness of models (dropout), add to the complexity and difficulty in interpretation. In spite of this, a simple ANN is constructed and trained on the data to provide metric comparisons.

## 2. Methods

### 2.1 Mouse model, tissue growth, and sample resection

To produce the samples for evaluating the spectral signature classification hypothesis of the spectrometer, *ex vivo* surgically explanted murine sarcoma samples were compared with otherwise healthy murine tissue in an equivalent anatomic location.

To determine an approximate number of mice necessary to attain a comparable classification accuracy to that of a surgeon using visual cues, an inverse power law regression was fit to validation accuracy results at various sample sizes for an artificial neural network, and then extrapolated to 90% classification accuracy [40]. This resulted in a prediction of approximately 500 total samples of healthy and sarcoma data- with reasonable interclass balance- needed to achieve comparable accuracy to a surgeon using visual assessment. However, a roughly 3:1 dataset ratio of sarcoma to healthy sample laser acquisitions was determined to be most similar to OR conditions, in which healthy tissue is rarely resected or available for *in vivo* photonic irradiation due to the potential danger it poses to patients [41]. Due to size differences between sarcoma and healthy tissue samples, and general availability of both tissue types, we also expected to encounter a roughly 3:1 ratio class imbalance between sarcoma and healthy tissue in this study, and thus determined an analytical pathway to address it, through use of weighted sampling for train and test datasets, and through evaluation of specificity and Area Under the Curve metrics. By doing this, conclusions from this study could be more readily translatable to human surgical data as well. In order to prevent spectroscopic recapture of any given point on a tissue sample, an estimate of 50–100 samples per tissue sample was determined to be fitting without laser spot overlap, and thus 5–10 mice were prepared for use in the study.

Once an approximate sample size was calculated, female sarcoma-bearing mice were generated in accordance with Duke University Institutional Animal Care and Use Committee-approved protocols (Duke University IACUC A025-21-01). The mouse genotype used was *LSL-Kras*^*G12D/+*^; *p53*^*Flox/Flox*^ and has been previously described [42]. Mice were obtained from Jackson Laboratory (Bar Harbor, ME). Soft tissue sarcomas were generated in the proximal portion of the medial or lateral gastrocnemius muscle as previously described [42]. Mice were manually examined for evidence of sarcoma growth or for likelihood of imminent morbidity. Once sarcoma tumors reached a verified size of 500 mm^3^, mice were removed from observation and prepared for sacrifice and tissue excision.

Mice were sacrificed using isoflurane gas and tumors were resected from left hind legs. Under supervision of mouse handlers, only blatantly visible tumor masses were excised with considerable under-resection at the tumor site, to circumvent the need for pathology staining, and to reduce likelihood of mislabeling. Lastly, ordinary hindleg muscle was removed from the opposite leg of each mouse in order to represent structurally similar healthy tissue control. All tissue samples were cleaned of hair and other particulate, though some samples contained small amounts of blood. This blood was not cleaned off since it was not noticeably substantial or actively accumulating on the surface of the tissue. Furthermore, any blood on the surface of the tissue allowed for more robust evaluation of the device signal capture capabilities in real intraoperative settings, in which considerable preparation of samples prior to imaging is not feasible due to patient safety and operative efficiency issues [43]. A total of 6 mice with sufficiently visible tumor growth were utilized for analysis; 4 other mice were disregarded, as they displayed questionable or visible lack of tumor growth to the required 500 mm^3^ size. All mice were sacrificed and imaged within 24 hours of sacrifice to minimize decay of tissue metabolic processes [44].

**Fig 1:**
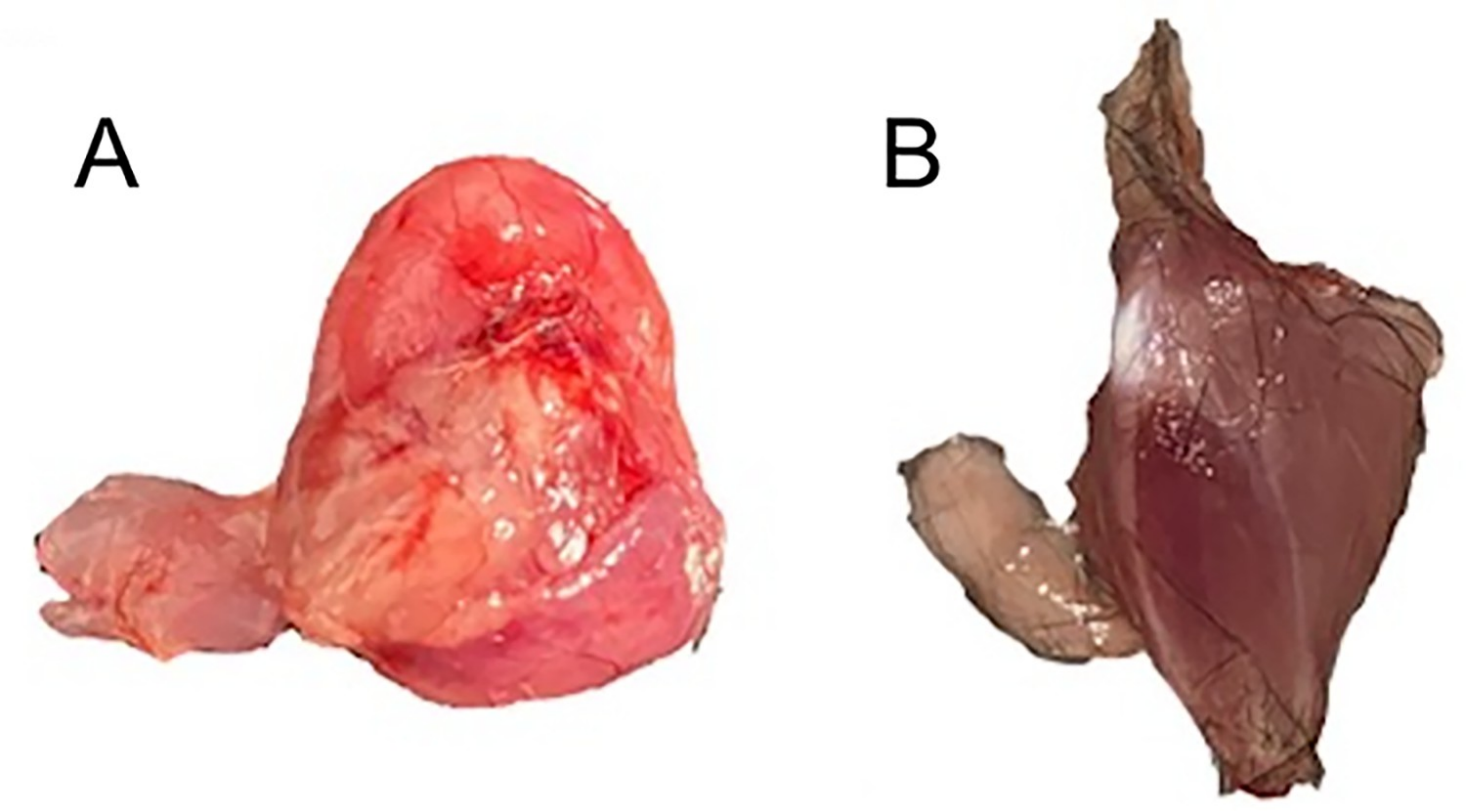
These figures visualize the two tissue sample classes: **(A)** pure sarcoma tissue, which was resected from murine hind legs, and is pink, globular, and nondescript in shape, and **(B)** analogous healthy tissue, which was resected from control hind leg tissue, and is visibly dark red, lustrous, and defined in shape. Samples are otherwise difficult to visually distinguish from one another.

Fig 1 demonstrates the typical tissue samples collected for both tissue classes. These and other similarly acquired samples were placed under the device for rapid collection of tissue spectral signatures, with no further preparation done.

### 2.2 Experimental set-up

**Fig 2:**
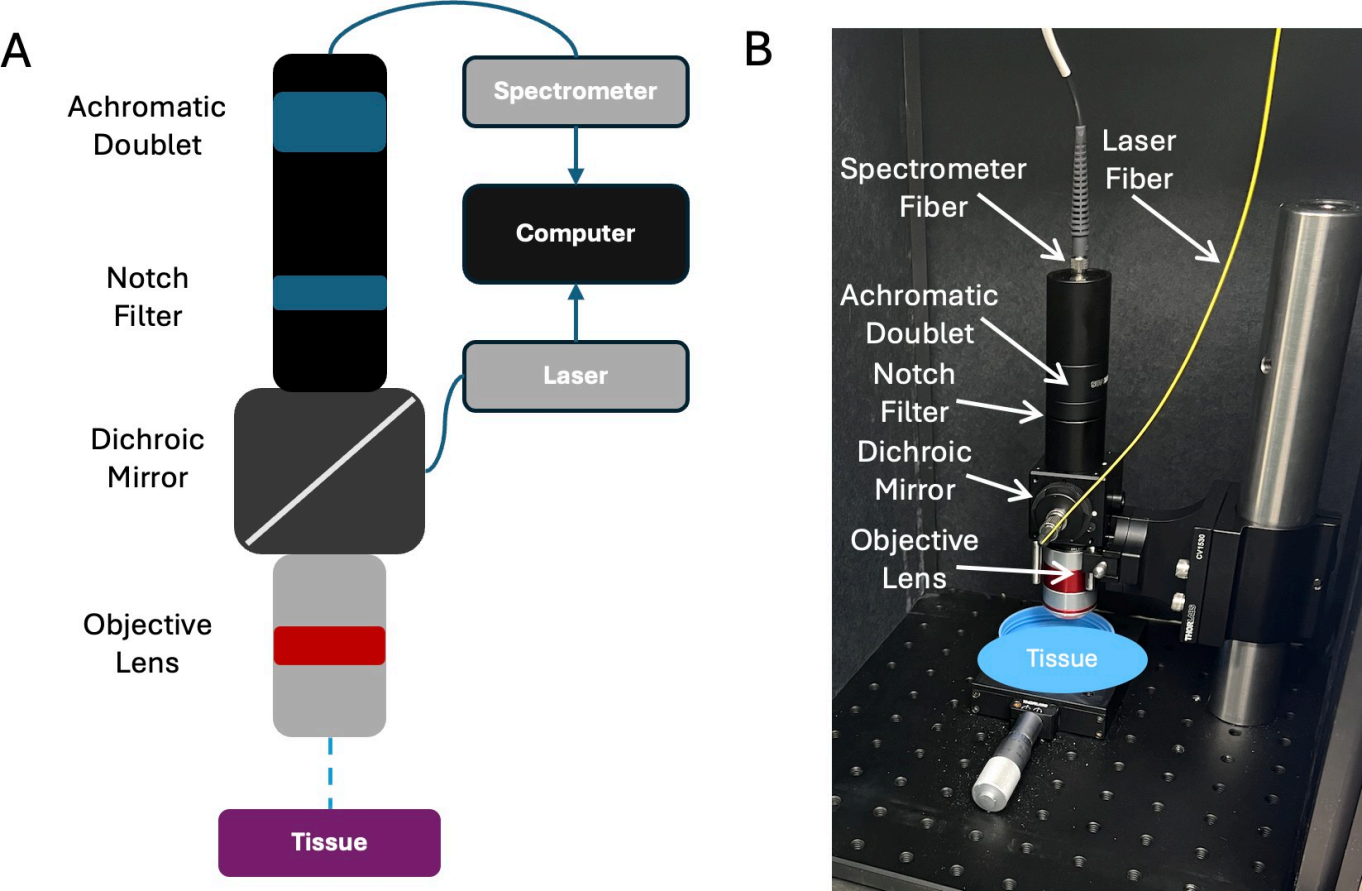
**(A)** Basic design schematic and **(B)** physical components of previously mentioned point-of-care autofluorescence device: excitation laser and detached spectrometer are intentionally designed for increased maneuverability in the surgical field.

The schematic shown in Fig 2 includes basic components used in the portable non-contact autofluorescence device. Various optical components focus a 405 nm laser source onto a sample, and reflected light is propagated through the same system anatomy to a CCD spectrometer for evaluation.

This point of care device used to collect data was previously developed by our group and described in detail in a previous paper [35]. The device is capable of epifluorescence through design, which allows for excitation and measurement of resulting emission from the sample. A 405 nm laser diode (Model LP405-MF300 from Thorlabs, Newton, NJ) is powered by a driver (Model CLD1010LP, Thorlabs, Newton, NJ). The excitation light is collimated, reflected by a dichroic mirror, then focused onto the sample by a 4X .2 NA apochromatic objective lens (Model TL4X, Thorlabs, Newton, NJ). The measured laser power for these experiments was 180 mW, with a 0.75 mm spot size and 0.5 s integration time. These wattage and time parameters were determined from previous studies to not have burn damaging properties to tissue [34].

Once sample tissue is excited, an autofluorescent emission pattern is formed and travels through the objective lens and a 425 nm long-pass dichroic mirror, and then subsequently focused into a fiber that leads to the spectrometer (Model CCS200, Thorlabs, Newton, NJ), using an achromatic doublet. The CCD spectrometer records wavelength-intensity pairs and this resulting spectral signature is saved to a computer. The entire signal acquisition process for the device accounts for nondestructive sensing; samples are not directly touched- which could cause deformation and damage- and no external dye is necessary to induce fluorescence, due to the device’s use case of autofluorescence, or endogenous fluorescence.

Before and during each laser exposure, ambient light was shut off in the experimental room to isolate the sample from external photonic effects. The sample and device were not completely shrouded, as is done in traditional spectrophotometer sample chambers, since complete shrouding of relatively unprocessed tissue is generally not possible in intraoperative settings.

### 2.3 Spectroscopic signature collection

Samples were placed 17mm from the objective lens according to its working distance. Once the device was optimally configured for data collection, each separate tissue sample was placed on a discrete clear plastic dish and set on the sample stage directly under the laser.

Ambient light was switched off prior to spectral collections by the device. Under low power laser light of less than 50 mW, the sample was positioned appropriately underneath the device. A Python program was then run to execute remote laser irradiation, signal acquisition, and data storage.

Between 50–100 spectral signatures were collected from each tissue sample, depending qualitatively on the size of a specific resected tissue sample. The sample was manually repositioned after every acquisition, and roughly 1 mm spacing was given between acquisition points to ensure no overlap between subsequent laser spots. Data from a given tissue sample were labeled in a csv file, according to both tissue pathology and specific mouse in the sample set. After this, the mouse carcass and tissue sample were discarded in accordance with Duke IACUC protocols and the next mouse was prepared for resection and tissue scanning. This process was repeated until 6 murine sarcomas and 6 healthy murine hindleg muscles were excised, cleaned, and scanned.

**Fig 3:**
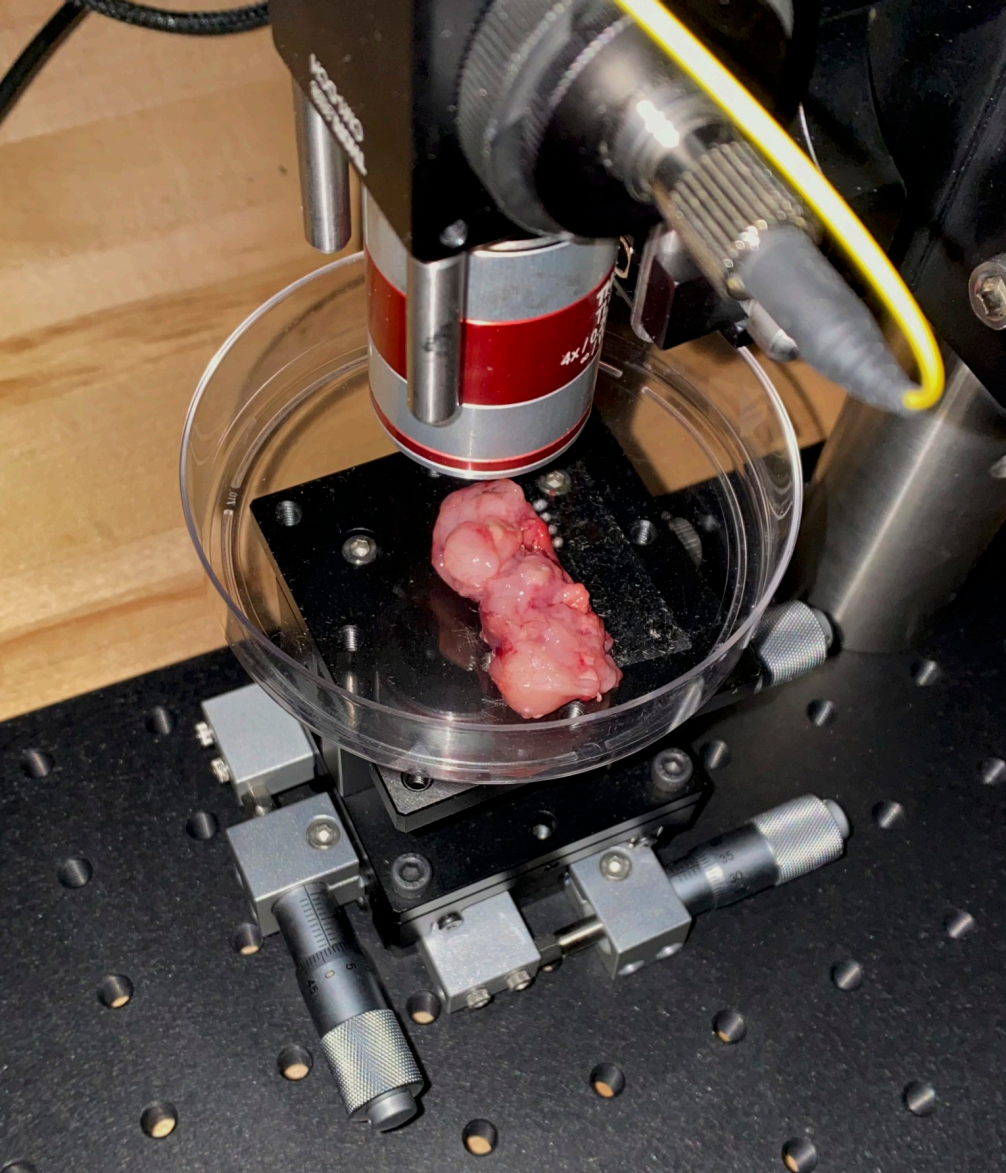
Specific configuration of autofluorescence device with freshly resected tissue sample prior to laser excitation and subsequent signal acquisition.

Fig 3 shows the sarcoma tissue sample positioning below the device apparatus. The sample is visibly large enough to collect multiple non-overlapping spectra for subsequent analysis, as the sample is substantially larger than the 1 mm spacing between spots. Also of note is the distance from the objective lens to the sample, which was not adjusted once acquisition of all samples began, so as to not affect intensity levels per spectra.

### 2.4 Data pre-processing

After acquisition, 513 spectral signatures were acquired, with a distribution of 395 tumor and 118 healthy data points prior to any data processing. The imbalance in data was due to the small size of healthy calve muscle samples excised, which caused the samples to have less adequate spacing for new surface imaging, according to the 1 mm spacing procedure. In general, healthy tissue was also less readily available from the analogous anatomical areas that sarcoma samples were excised from, preventing equality in class representation.

Various data pre-processing steps were performed to ensure each signature was not affected by poor signal acquisition or signature creation. Firstly, due to intense reflection of 405 nm light into the spectrometer from the sample, in spite of dichroic and notch filtering components, a high-pass cutoff of 445 nm was used to consider only wavelength measurements reasonably unaffected by the artificial light intensity measurement, including rolloff from the high-intensity 405 nm excitation peak. This cutoff was intentionally chosen to be the same threshold wavelength as that of the dichroic mirror used in the optical setup, which has a 90% attenuation factor at 445 nm. A hard lowpass cutoff of 750 nm was also utilized, as beyond this was determined to be a wavelength region outside of the expected range for important metabolites at 405 nm excitation, and any differences in this region were attributed to system noise.

Additionally, to eliminate physically nonviable data, any spectral signature with an average intensity of less than .005 was disregarded. This threshold was used to indicate data with low signal-to-noise ratios brought on by poor sample positioning. After this data clean-up, 511 spectral samples remained, with a distribution of 393 tumor and 118 healthy spectral signatures. Next, data was smoothed by means of a moving average filter with a window of 10.

Lastly, data was normalized with respect to the 500 nm wavelength, as previous studies have shown that the biomarkers described by the Warburg effect are most evident around this wavelength when using 405 nm excitation [30–31]; by normalizing with respect to each spectrum’s intensity at the 500 nm wavelength, special attention can be paid toward the wavelengths in this area, in order to classify based off the Warburg effect. This specific method also helps to ensure that, during automated classification, shape of signature is considered in addition to overall intensity in classifying tissue according to pathological state. This is more accurate and robust, as biologically-informed classification based on pure average intensity difference can be erroneous due to variations in working distance to the sample, angle of incidence for the laser, color of individual tissue samples, and other extraneous, non-physiological issues.

### 2.5 Statistical analysis

Statistical analyses were conducted using GraphPad Prism 9.5.3 (GraphPad Software, San Diego, California), in order to compare specific fluorophore content for each tissue class and determine significant differences according to pathology. Using trapezoidal integration within the full width half maximum window referenced in Table 1, area under the curve values for each spectrum were calculated for bound NADH, free NADH, FAD, bound porphyrins, and neutral porphyrins. The area under the curve scores for each fluorophore type were compared using a two-tailed difference of means Mann-Whitney test between sarcoma and healthy tissue, and using an alpha significance value of 0.05. The data were not found to be normally distributed by the Shapiro-Wilk test under a significance value of 0.05, and therefore the non-parametric Mann-Whitney U-test was selected for use in this study.

### 2.6 Classification pipelines and methods

After pre-processing, data was fed into several dimension reduction and classification pipelines for a comparative study of performance in sarcoma classification. Moreover, a basic comparison of neural networks versus traditional prediction algorithms was performed, to investigate the necessity of the computationally intensive and “black-box” neural network learning framework for this application.

Data was feature engineered through PCA in order to extract or reduce dimensionality. Specifically, the analysis determined that 95% of the variance in the dataset was explained with 5 PCs. Therefore we used 5 PCs our model, which was an appropriate trade off between model complexity and performance, and is standard practice in PC analysis. Feature engineered data was inputted into 3 different classification algorithms: KNN, LR, and SVM. Various evaluation metrics were analyzed to determine the best classifier for the study’s specific dataset, including accuracy, Area Under the Curve (AUC), precision, recall, F1-Score, and specificity. Lastly, raw data was also inputted into an artificial neural network (ANN) and similar metrics were obtained, though data were not feature engineered prior to input because the neural network conducts this process on its own.

Each method, other than the ANN, passed through a similar nested Grid Search cross validation pipeline, in which certain hyperparameters were tuned to the specific dataset using a number of initial guesses. Grid Search is a computational method in which a list of different hyperparameters is predefined by a user, and each possible permutation of hyperparameters is trained on the dataset and subsequently validated on a holdout dataset. The combination of hyperparameter values with the best performance, as determined by the user, is then retrained on the entire training set. We chose to perform Nested Grid Search to add robustness to pipeline results. Nested Grid Search involves Grid Search within a training fold- created through K-Fold Cross Validation- and subsequent choice of hyperparameters, repetition across all K folds, and then performance comparison of the best hyperparameter-optimized model from each fold before choosing the ultimate model. This training method is explained in detail in literature [45].

For purposes of our study, the total dataset was split in an 80:20 manner for training and testing. Next, the training set was divided twice according to K-fold validation, with outer folds divided into fifths, and inner folds, where Grid Search took place, divided into fifths as well. This ensured that tuned models were not being evaluated on previously seen data. This Nested Grid Search process allowed generalizability of hyperparameter-optimized models to be evaluated, as each training dataset was slightly different depending on the specific K fold.

Parameters optimized for SVM with a Radial Basis Function kernel were C and gamma. C is a penalty for proximity of support vectors to a decision boundary, and gamma is a kernel coefficient used to describe the spread of the kernel. Optimal hyperparameters for this dataset and problem were determined to be .001 for gamma and 5 for C.

For LR, C, the type of solver, and the type of penalty were optimized in the Grid Search process with attention to accuracy performance. C is the same factor as in SVM and the solver is the specific method used for optimization and minimization of error. The type of penalty includes L1, L2, or no penalty and these terms are used toward regularization of the model to prevent overfitting. C for this dataset was best determined to be .001, and the best solver was newton-cg, while the L2 norm was deemed the best penalty term.

For KNN, number of neighbors, distance calculation, and weighting method were considered in the Grid Search, with attention paid to accuracy in determining suitable hyperparameter combinations. Optimal number of neighbors was determined to be 5, and the weighting method was determined based on distance and found to work best under the Manhattan distance metric.

All hyperparameters optimized in the Grid Search for each model are specified collectively in Table 2 below, with specific values included for each hyperparameter.

**Table 2:** Specific hyperparameters optimized for each non-ANN model in the Nested Grid Search process.

SVM (with RBF)	LR	KNN
**C:** 0.5, 1, 5, 10, 30, 40 **Gamma:** 0.001, 0.005, .01, 1/(num. of features), and 1/(num. of features* dataset variance),	**C:** 0.001, 0.01, 0.1, 1 **Solver type:** ‘newton-cg’, ‘lbfgs’, ‘sag’, ‘saga’ **Penalty type:** L1, L2, none	**Number of neighbors:** 5, 7, 10, 15, 20 **Distance calculation method:** Manhattan, Euclidian, and Minkowski **Weighting method:** distance-based or uniform

A separate ANN framework was created with the following structure: 3 fully connected layers with ReLu nonlinear operators interpret the cleaned spectra and fit them into 2 end neurons, corresponding to tissue class. There is appreciable reduction in dimensionality at every new layer, until a binary outcome is achieved, which represents the classification task at hand.

Parameters used for ANN training are as follows:

**Table:** 

ANN Model Parameters
Epochs: 35–100 Batch Size: 4 Learning Rate: .001 Number of Input Features: 1323 (trimmed wavelength range) Dense(nodes = 128, activation = relu) Dense(nodes = 64, activation = relu) Output: Linear(nodes = 2), Activation = None, (number of Output Classes) Optimizer: Adam Loss: Categorical Cross Entropy

The ANN was trained and validated five times and scores were averaged to depict a more robust evaluation of the pipeline, similar to the architectures that were subject to nested Grid Search Cross Validation. PCA was not used for the ANN because neural network-based classification inherently handles dimensionality reduction, and we did not want to risk losing vital feature information or assume a linear relationship between features prior to classification by the ANN.

Additionally, ROC curves and learning curves were generated for each of the four methods as an additional means of analysis. The ROC curves provide insight into the models’ performance across the range of classification thresholds between 0 and 1.0 and visualizes the balance between true-positive rates and false-positive rates [46]. It also affords us the ability to calculate the area under the curve (AUC) score, which is an overall metric of performance for the model across all classification thresholds, with an AUC of closer to 1.0 indicating better performance. The learning curves on the other hand visualize the training performance of the model on the training and validation datasets against the number of training iterations [47]. These plots are useful in diagnosing model issues, e.g. model overfitting, and determining at which iteration training should be stopped to avoid such issues. Both ROC and learning curves were used to assess the models’ performance and tune the training process.

## 3. Results

### 3.1 Raw data and initial observations

**Fig 4:**
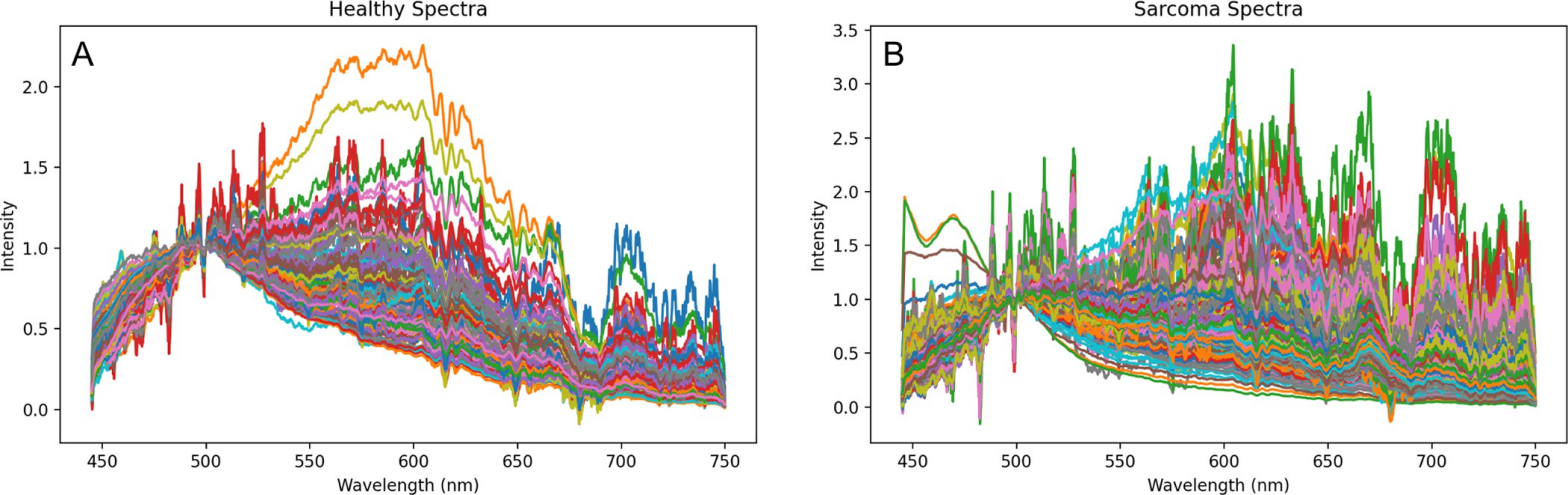
All spectra for **(A)** healthy and **(B)** sarcoma tissue in spectral regions of interest after 500 nm-pin normalization. Healthy spectra appear to generally have more muted intensities after 500 nm, relative to sarcoma spectra.

Spectral signatures acquired from each class of tissue with the point-of-care fluorescence device were plotted according to the experimental set-up. This yielded the following set of healthy and sarcoma tumor tissue spectra, outlined in Fig 4A and 4B with appropriate cutoffs, smoothing, and normalization at the biologically relevant 500 nm wavelength region.

**Fig 5:**
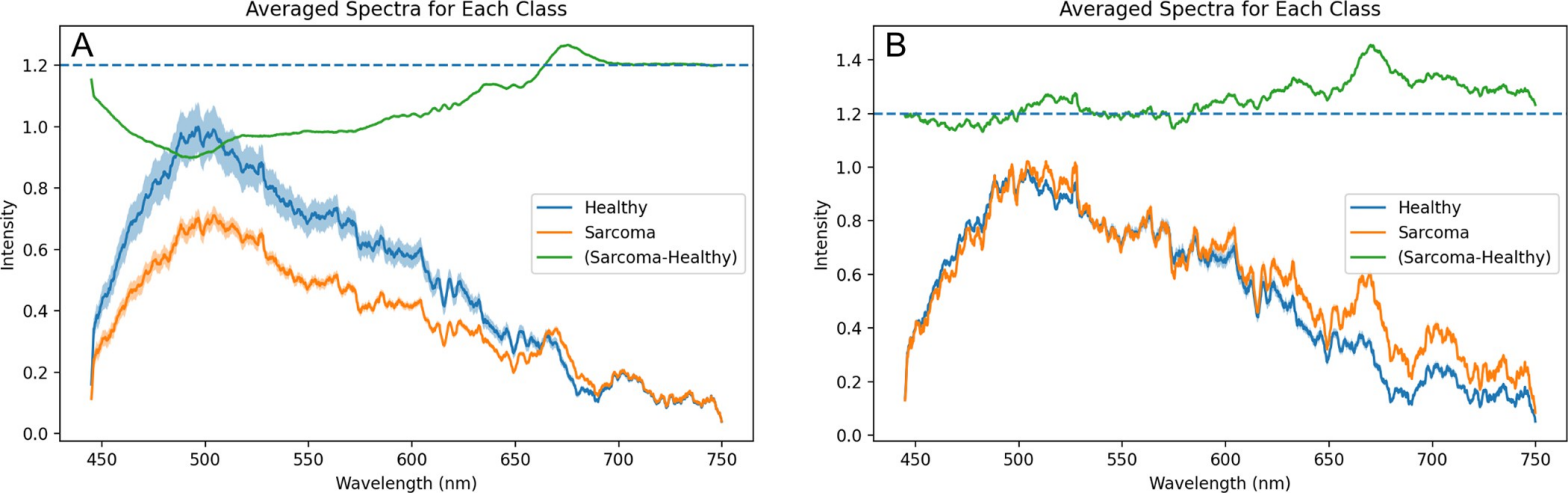
Average spectra for both tissue classes, with standard error regions. Differential normalized fluorescence curves, or the difference of sarcoma from healthy averaged spectra, are shown in green, arbitrarily positioned at 1.2 for ease of visualization. **(A)** Averaged spectra for each tissue class without individual spectra normalization. Spectra are scaled between 0 and 1.0 through division by global maximum intensity. Standard errors are provided as shaded regions around average spectra. Spectra are observed to significantly deviate in intensity between 450 and 650 nm, but converge around the 700 nm region. **(B)** Normalized spectra with standard error shaded regions. Every spectra is normalized prior to averaging by class, using 500 nm intensity division per spectra, then averaged data is all divided by the global maximum intensity.

As is seen in Fig 4A and 4B, it is difficult to visually ascertain major differences, aside from some noticeable unique peaks in the sarcoma spectra. Thus, all spectra were afterwards averaged and presented in a single plot with standard errors (Fig 5). To generate data for Fig 5A, no normalization was done to individual collected spectra; rather, all raw spectra in a given class were averaged, and the resulting two averaged spectral class curves were divided by the global maximum average intensity value, which was the maximum value of the averaged healthy spectra curve in this study. The differential normalized fluorescence, or the difference between the averaged sarcoma spectra and healthy spectra, is shown as a green plot with all values arbitrarily elevated by 1.2 intensity units for ease of visualization.

Fig 5A demonstrates that healthy spectra have higher intensities of back-reflected light than sarcoma spectra from 450 nm to roughly 660 nm. Also, notably, there is a prominent and broad observed differential fluorescence intensity peak at 475 nm. Around 660 nm, spectra begin to behave similarly, however there is a large positive differential normalized peak at 675 nm, contradictory to the negative differential fluorescence values in preceding wavelength regions. Standard errors within spectral classes become smaller as wavelengths get larger and further from the 405 nm excitation pulse.

After each individual spectral acquisition was normalized, we generated each class’ average spectra to highlight what type of differences would realistically be utilized by the classifier architectures (Fig 5B). Fig 5B is provided to address the potential for confounding effects that may be apparent in Fig 5A, such as the effects of ambient light, working distance, and other non-physiological factors, which can contribute to the spectral curve shapes and intensities.

Notably, there are evident peaks in the Fig 5B differential normalized fluorescence curve (shown in green) at 480, 520, 575, 650, and 671 nm. Including standard errors in the Fig 5B plot, these differential regions correspond to significantly non-overlapping spectral regions, implying statistically significant differences in normalized intensity of reflected light from diseased and healthy tissue at these wavelengths, and validating the statistical analyses run previously. Fig 5 demonstrates that spectra of a given class are roughly similar in waveform pattern, albeit with minor differences in intensity, contributing to the small standard error in the normalized spectra. Fig 5A, in spite of no per-spectra normalization, shows a visibly evident difference in cancerous and healthy tissue, with the average cancer spectroscopic signature being muted in comparison to the healthy spectra. Fig 5B, however, represents the implementation of the 500 nm-based normalization, used to adjust spectra for machine learning model input. Normalized spectra look more similar in Fig 5B than in Fig 5A, however, in Fig 5B, inferences can be made on metabolic tissue differentiators as per the Warburg effect, rather than structural tissue properties or extraneous data acquisition schemes that could confound conclusions from Fig 5A.

Out of the six emission regions tested, five had statistically significant results according to the analytical procedure outlined in Methods 2.4. Free NADH (445 nm—529 nm) was significant (U = 18891, p = 0.0001). FAD (506.5 nm—581 nm) was significant (U = 18950, p = 0.0026). Similarly, both basic porphyrins (577.5 nm—602.5 nm) and neutral porphyrins (617.5 nm—642.5 nm) were found to be significant (U = 19097, p = 0.0033 for basic porphyrins, U = 16632, p < 0.0001 for neutral porphyrins). Interestingly, the emission region between 658.5 nm and 683.5 nm was also found to be significantly different (U = 9509, p < 0.0001). The region for Bound NADH (469 nm—533 nm) was not found to be significant (U = 21059, p = 0.1304). This conclusion supports that the two spectra differ, as corroborated in Fig 5A with the lack of overlap of standard error boundaries in these regions, and visible peaks in the Fig 5B differential normalized fluorescence curve.

### 3.2 PCA

**Fig 6:**
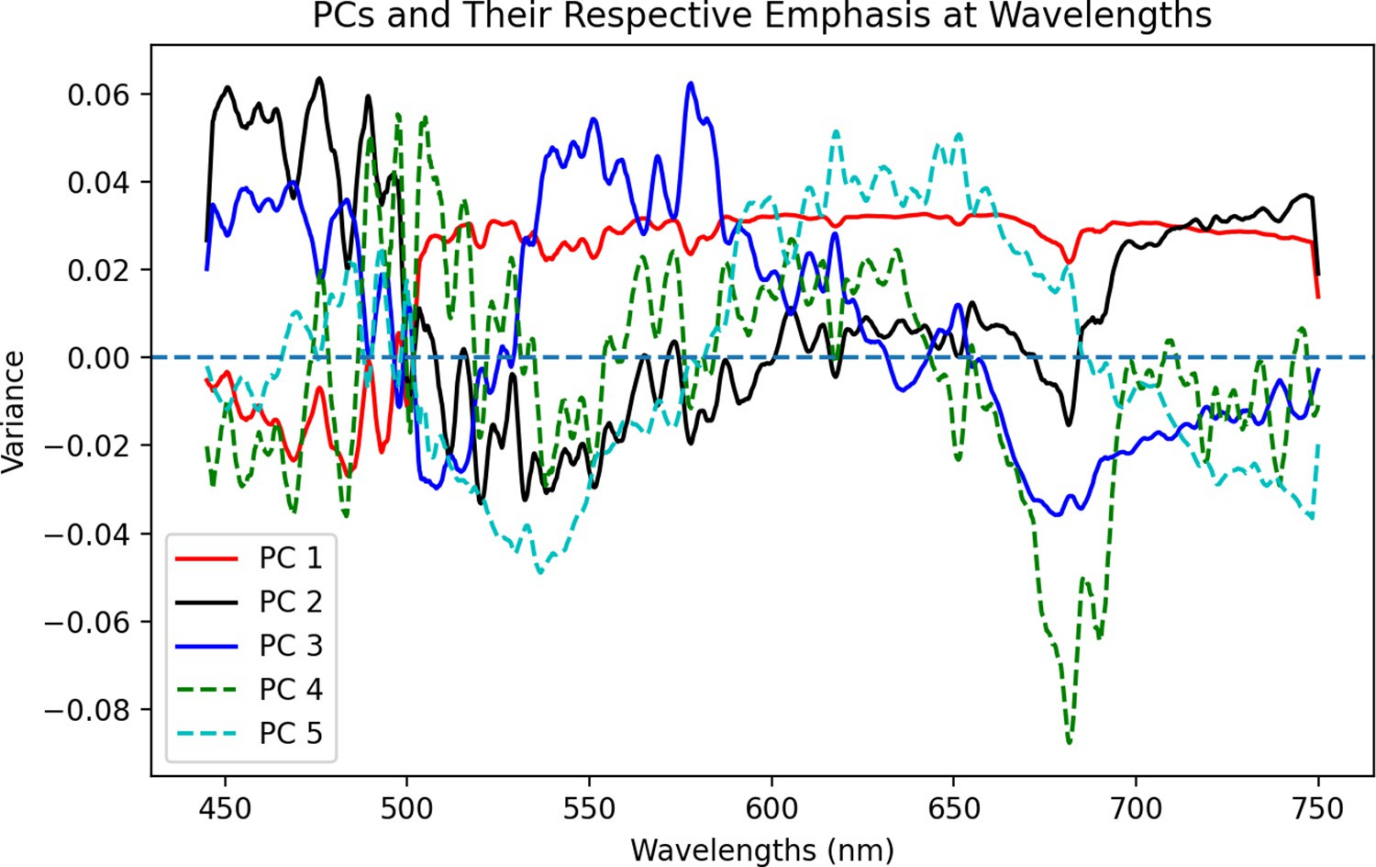
Plot of 5 most important principal components (PCs), defined as those PCs making up 95% of total dataset variance together, and their respective variance intensity at each wavelength. The first 3 PCs, representing roughly 90% of total dataset variance, are shown as solid line plots, while the final 2 PCs are shown as dashed line plots.

After conducting PCA on the 1323 input feature wavelengths to reduce dimensionality, the top 5 PCs, representing 95% of the overall dataset variance, and their corresponding basis spectra were plotted to illustrate which wavelength regions made up a large portion of a given PC’s variance, in a quantifiable manner. Due to the linear manner of conversion from high-dimensional spectra to extracted PCs, the conversion matrix can be determined through an inverse matrix operation. This transformation, which displays the wavelength range versus the variance at a wavelength for each PC, is shown in Fig 6. Because the 480, 540, 590, 630, and 670 nm regions display some of the largest variance for PCs 1–3, which together account for approximately 90% of the total dataset variance, it is expected that any classification architecture using PCA will heavily depend on these regions in particular to draw decision boundaries and group data. The final 2 PCs, representing roughly 5% of the total dataset variance, are shown as dashed line plots to illustrate the total extent of feature importance used in the classification task. Though they are less important to the classification task than PCs 1–3, PCs 4 and 5 still contribute to overall optimal classification.

### 3.3 Classifier performance comparison

After PCA, the 3 machine learning models and grid search cross validation pipelines were implemented on the data. The 5-fold averaged ANN was also trained and evaluated on the data for performance comparison, without PCA or Grid Search, according to the specific architecture outlined in Methods.

Table 3 shows the results from nested cross validation for each pipeline using 5 PCs; above this number of PCs, the models begin to perform much worse, likely due to overfitting from the capture of device or process noise in less representative PCs; 5 PCs provide the best classification performance on held-out test sets. Also included are the averaged ANN results from 5 consecutive training sessions to account for the difference in output metrics due to stochastic gradient descent for every model instantiation.

**Table 3:** Comparison of performance between the machine learning pipelines for tissue classification. For precision sensitivity, and F1 score values, both positive class scores and macro scores are provided to address class imbalance, with positive class scoring on top and macro scoring on bottom of each cell.

Model	Accuracy	Specificity	Precision	Sensitivity or Recall	F1 Score
PCA-KNN	.88	.67	.90 .85	.95 .81	.93 .83
PCA-SVM	.87	.71	.91 .83	.92 .82	.92 .82
PCA-LR	.93	.75	.93 .94	.99 .87	.96 .90
ANN	.91	.79	.94 .88	.95 .87	.94 .88

It is important to note that, since a substantial class imbalance was intentionally created, output metrics must take this imbalance into account. For precision, recall, and F1 score, two numbers are provided for each model: the first represents the single-class metric value for sarcoma classification, and the second number represents the macro-average of the classification task, or the value for which both class scores are weighted equally and scores are averaged, treating both healthy and pathological tissue as equally important to classify correctly, even though healthy tissue is less represented in model training. This is naturally expected to cause lower evaluation metrics.

As Table 3 illustrates, using a computationally intensive hyperparameter optimization and multi-layered cross-validation, PCA-Logistic Regression performs well, with an accuracy of 93% and specificity of 75%. LR had higher macro scores for precision, recall and F1-score compared to the other models, though the metrics are lower than if simple micro-classification of the positive class were used to evaluate models instead. The ANN also performs quite well with an accuracy of 91% and optimal specificity of 79%, however insight into the specific weight optimization within its architecture is unknown due to nonlinear operators between ANN layers, and thus similar physiological implications made from PCA, as in Fig 6, are not possible. Activation maps would not provide an accurate picture of the basis for feature preference due to the presence of nonlinearities like ReLu between ANN layers, and thus are not displayed here. It is important to note that SVM also performs well, albeit slightly worse than LR in the Table 3 metrics. Specificity of 71% is comparable to LR, however most other metrics are noticeably worse than both LR and ANN. KNN also performs with high accuracy, however specificity is very low at 67% and macro scores for precision, recall, and F1 score are lower than all other pipelines.

Because more than 3 PCs are necessary to explain over 95% of the data variance for this particular problem, it is not possible to show an accurate visualization of the decision boundary created by SVM or LR. A boundary hyperplane using 2, or even 3 dimensions would achieve poor training accuracy, and thus not predict well on the test set.

**Fig 7:**
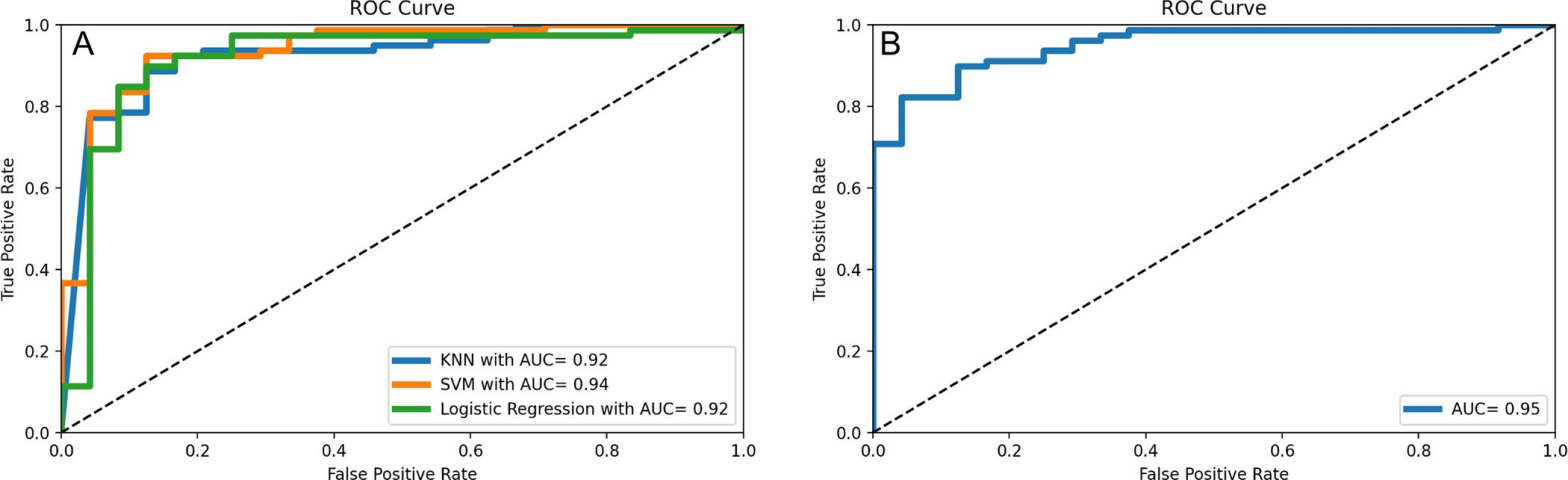
**(A)** ROC curves show relative performance of each non-ANN machine learning method, **(B)** Aggregated performance ROC curve for ANN with 35 training epochs. Area Under the Curve (AUC) metrics are provided in the figure legends to demonstrate interaction of false and true positive rates.

Additionally, Receiver-Operator Curves were created for the best performing model within a given model class, and these are shown in Fig 7. As can be seen in Fig 7A, SVM has the best AUC score at .94, indicating the lowest rate of false positive increase for each marginal increase in true positive rate; this is displayed by its ability to reach the upper-left corner of the plot better than the other two classes, and subsequently integrate to the highest AUC score. Depending on thresholding, this would indicate SVM performs best; because of the dataset imbalance, AUC is a highly important metric that quantifies performance irrespective of weighting. Fig 7B shows the standalone ROC curve for the ANN, which is also relatively well-performing with an AUC of .95. It performs comparably to the best performing SVM case, demonstrating its ability to correctly classify tissue as cancerous without over-classifying.

**Fig 8:**
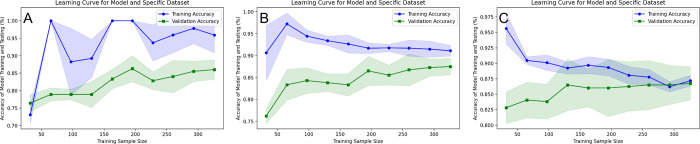
Learning curves for 3 non-ANN models in order: **(A)** KNN, **(B)** SVM, **(C)** LR. These learning curves are generated through use of 90% of the total collected spectra for this experimental study. From this data subset, 5-fold Cross Validation is conducted on each train sample fraction that is generated from a list of increasing training size proportions (0.1, 0.2, 0.3, etc.), and the average of the resulting 5 training scores is provided as a single point with standard deviations shown as shaded regions. The remaining fraction of data is treated as validation data, and its related averaged accuracy and standard deviation is provided in the green curve.

Lastly, learning curves were created for each machine learning pipeline to compare and understand the bias and variance properties. This is shown in Fig 8. All 3 models have reasonably low bias, however LR and SVM appear to slightly overfit with smaller datasets, as indicated by their decrease in accuracy as instances increase in the training process and models become more robust. Variance is especially low for Logistic Regression and SVM, since the distance between validation and training accuracies is minimal in their trends. This further corroborates the use of LR and SVM, as they demonstrate high accuracies, but also generalize well to holdout data.

**Fig 9:**
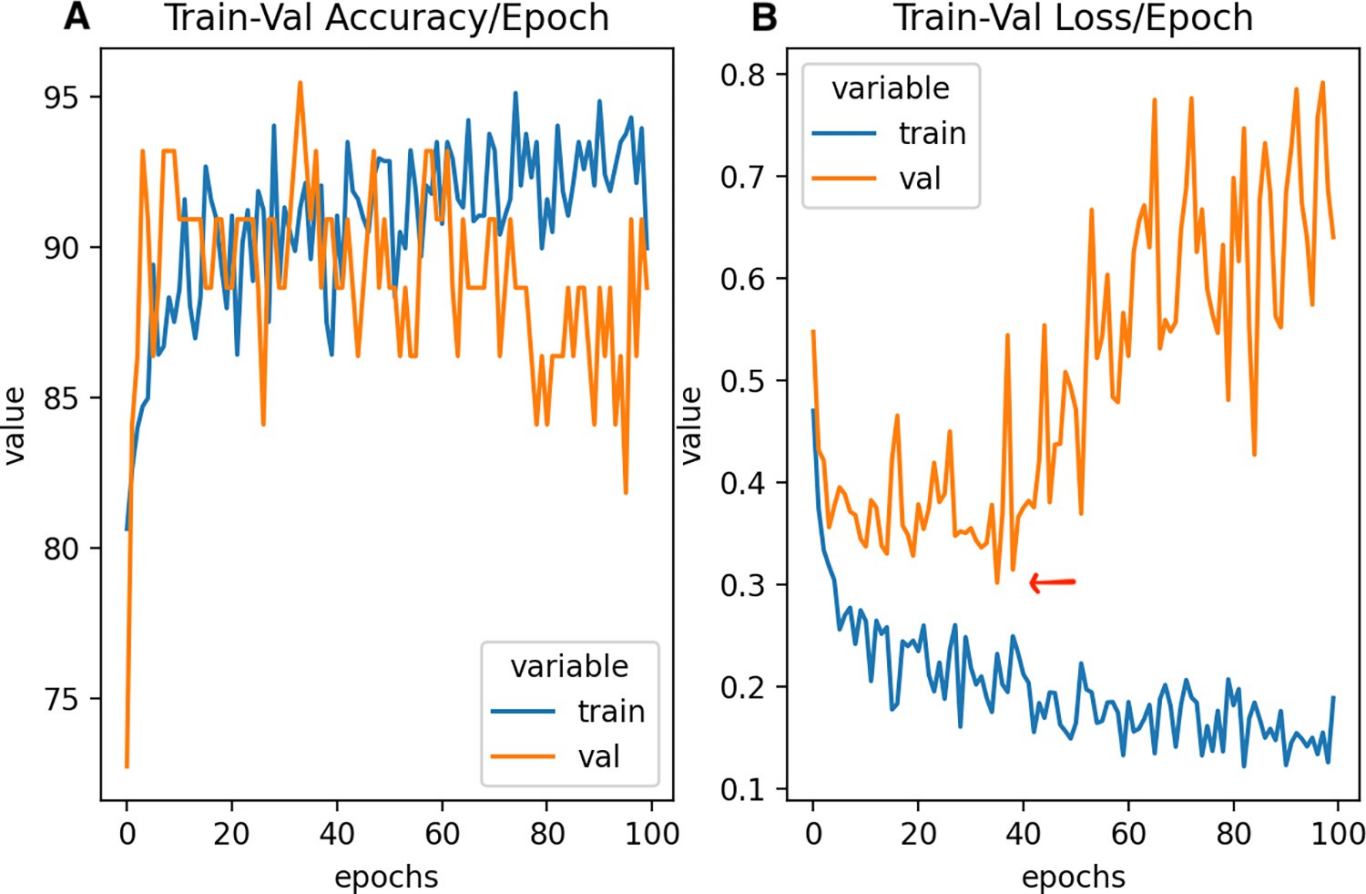
**(A)** Accuracy curve for ANN training over 100 epochs and **(B)** loss curve for ANN training over 100 epochs. Optimal training epoch number is identified as 35 epochs, as indicated by the red marker.

Fig 9 illustrates an analogous metric for the ANN, with loss curves and training accuracy as a function of epochs. As can be seen, the optimal number of epochs for training is approximately 35, as validation loss begins to increase drastically after this, indicating substantial overfitting of data. This low number of epochs needed for training is reflective of the overall small size of the dataset, ease of binary classification, and relative ease in separability for the two classes. Additionally, it can be seen that loss decreases and stagnates up to the 35 epoch mark. Accuracy of validation and train sets are relatively equal as well, indicating low variance between sets and overall similarity in hold-out spectral samples. It should be noted that even a simple neural network architecture, as shown here, may perhaps be too complex and potentially unnecessary for classification of cancerous and healthy tissue. Simpler methods, as shown above, achieve similar metrics of performance in a much more interpretable- and much less resource intensive- manner.

## Summary and discussion

Autofluorescence has long been hailed as an effective tool for characterization of tissue, including pathologic tumor tissues, with implications for oncologic surgery. The use of endogenous fluorophores, which are electron-carrying chemical complexes important to cellular metabolism, can help to differentiate cancerous from healthy tissue. Moreover, in an intraoperative setting, a fluorescence-capable device, which can collect spectral signatures rapidly and in a noncontact manner, can serve as an assistive tool to surgeons as they precisely operate at ambiguous tumor-healthy tissue boundaries. In this work, we excised sarcoma tissue from freshly sacrificed mice and imaged the samples under a point-of-care laser-spectrometer system adapted for use in neurosurgical operating rooms, producing spectral signatures that exhibited stark differences between cancerous and healthy tissue classes.

Sample spectra were collected multiple times (approximately 50 laser acquisitions per sample), with each spectra collected at a different point on a given sample. Though samples were not collected in multiplicate fashion, our method of collection, in addition to our choice to not consider collected spectra with an average filtered intensity below a .005 threshold, ensured that the device was collecting data correctly, and only physiologically relevant data was evaluated. Sarcoma samples were visually confirmed by a mouse pathologist to be cancerous, and excisions were done conservatively- under-resected well within the identified bounds of the embedded tumor- to ensure each sample was of only a single class type.

In total, 511 spectra were utilized for model training, 393 sarcoma and 118 healthy tissue spectra. This class imbalance was accounted for in classification with stratified and weighted sampling, as well as through special attention given to specificity and AUC metrics. Though the sarcoma spectra outnumber the healthy tissue spectra in a roughly 3:1 ratio, models still generalized well to testing sets, and metrics other than accuracy helped to explain model performance in spite of the imbalance. Differences between cancerous and healthy spectra were also substantial enough to warrant visualization in spectra patterns and intensities. In spite of this, certain issues did arise due to the imbalance, so we may explore more balanced data collection in the future. It is important to note, however, that healthy tissue is not usually available for collection in human surgical procedures; especially in delicate areas of the body, as in the case of spinal sarcomas or brain tumors, much care is taken to not remove or overly perturb healthy tissue at risk of harming critical collateral structures [48–49]. While control tissues can occasionally be obtained from non-oncologic surgeries where normal tissues are being removed (for instance, collecting normal brain tissue specimens removed in standard epilepsy surgery), comparable healthy control tissue is not always available in all oncologic surgery subtypes. [50–51]. This study, though conducted on mice, is more representative of conditions in intraoperative settings, in which there is a dearth of healthy tissue.

Using nested grid search hyperparameter-optimized and multifold cross-validated machine learning architectures, we were able to rapidly achieve classification scores of around 90%, which introduces the potential to aid pathologists and surgeons in terms of accuracy and speed. Moreover, with principal component analysis and the LR and SVM models, we were able to outline specific weights corresponding to wavelengths with high inter-class variance that aided in the classification process. Namely, free NADH, FAD, and basic and neutral porphyrins, important biomarkers in the cell metabolism process, are markedly different in quantity between the two classes, as shown by the overall muted intensity of sarcoma tissue, as well as their significant difference from healthy tissue at their hallmark wavelengths of 487, 544, 590, and 630 nm, in Fig 5A and 5B [30–31]. The broad 475 nm peak in the Fig 5A differential normalized fluorescence curve represents the known wavelength of emission for free NADH electron carriers at 405 nm excitation. Together, these data and corresponding statistical comparisons, indicate the presence of a significantly different number of NADH carriers in healthy tissue cell cytosol than in sarcoma tissue, as outlined by the Warburg effect (Fig 5B), as well as the contribution of increased scattering- and subsequently reduced reflection into the device spectrometer- in pathological tissue due to inhomogeneous or structured cell shape (Fig 5A). Fig 5A and 5B follow the literature trend for soft tissue spectra with 405 nm light excitation, and both are important to understanding the classification problem and underlying physiological principles [30–31].

Fig 6 further corroborates the role of differential contribution of fluorophores in tissue classification. The PC emphasis plot demonstrates that NADH, FAD, and porphyrins play pivotal roles in the creation of variance necessary for effective classification, as seen by high variance at the 480, 540, 590 and 630 nm wavelengths, as well as the previously seen 671 nm range in Fig 5A and 5B. Because the important wavelength regions of Fig 6, which represents 95% of dataset total variance, closely mirror those of Fig 5B as areas of high variance, it can be concluded that linearity of PC conversion is maintained, and adequate class-differentiating information is preserved with 5 PCs (95% of dataset variance). The PC emphasis plot of Fig 6 is necessary to visualize differences and translate back to biological processes.

Comparison of SVM and LR with ANN using Table 3 shows the relative similarity between the algorithms in performance, however SVM and LR are highly interpretable and likely more applicable for future purposes. It is difficult to ascertain whether the ANN high classification rate is due to the model’s focus on areas of high variance like wavelength regions reflective of NADH and FADH presence, or whether the model is classifying on arbitrary or artificial differences that result from the data collection process’ inherent noisy nature. With an accuracy of 93% and specificity of 75% for LR, it is more conducive to application in intraoperative settings, for which the device is intended. This LR preference over SVM can likely be attributed to the nature of the data. The two models usually perform comparably, however, Logistic Regression is preferred in situations where data is linearly separable. Though it is not possible to visualize due to the required five-dimensional feature space from PCA, there exists a linear boundary hyperplane that can separate the two classes easily, compared to SVM, which tends to work better for more overlapping datasets that require a non-linear decision boundary and make use of the RBF kernel.

SVM’s superior AUC, as seen in Fig 7, also may indicate its applicability in intraoperative settings, as it accounts for the potential of false positive classification at various thresholds. It is important, especially in an intraoperative setting, to diminish false positive likelihood, as this could lead to erroneous regional diagnoses and a potential indication to remove healthy tissue. The relative variability in LR and SVM model performance in Fig 8 learning curves as training size increases can likely be attributed to the class imbalance and how individual spectra are randomly assigned to a training or validation set; this is not reflective of poor classification performance, as it is more generalizable than a small training set. While other studies have shown the usage of artificial neural networks in a diagnostic device, our method shows an interpretable machine learning framework in which to classify tissue without compromising ability to further verify underlying physiology.

The Warburg effect describes how tumor cells exhibit larger quantities of electron carriers like NADH and FADH in their cytosol due to their over-consumption of resources and resulting inability to engage in aerobic steps of cellular metabolism. Because of a lack of proper vasculature prior to metastasis, these tumors also exhibit lower numbers of porphyrins, which are precursors to hemoglobin, an important oxygen-transporting molecule in red blood cells. Our sensing and analysis methods show a clear significant difference in reflected intensity at wavelengths known to represent these three endogenous fluorophores, as shown in Figs 5B and 6 and related statistical testing in Results. This can be attributed to the different number of endogenous electron carriers in sarcoma tissue cell cytosol, emitting distinct photonic intensities into the device spectrometer for subsequent spectral creation, and thus resulting in significantly different intensity measurements compared to those of healthy tissue, which contain less electron carriers and more porphyrins. In addition, the highly scattering nature of cancer tissue, due to its inhomogeneous cellular form, causes much less light to diffusively reflect back into the spectrometer, causing an overall muted intensity in sarcoma tissue, as seen in Fig 5A, and demonstrated in previous studies [52]. The four wavelength peaks in Fig 5B at 480, 520, 575, and 650 nm correspond with values found for bound and unbound NADH, unbound FAD, and porphyrins [30–31]. However the substantial peak at 671 nm is unexplained, though it is shown in other studies using differential normalized fluorescence-based tissue characterization [14,53]. We intend to investigate this particular difference in intensity between sarcoma and healthy tissue in future studies, as this was a contributing factor to the high classification performance of our machine learning algorithms, as further demonstrated in Fig 6.

It is also important to note the conditions under which samples were collected, which contributed to the overall noisiness of data and apparent difficulty in classifying tissue types above 90% accuracy. In regular spectrophotometer-based studies, samples are often prepared for lab usage prior to spectroscopic capture: cells are cultured, diluted in purified water, and mixed with various buffers that can artificially enhance classification ability and change *in vivo* tissue structure. Moreover, samples are covered in complete darkness prior to excitation by laser, preventing interference from any ambient light. In our study, samples were exposed to more ambient light during recording than would be possible in a traditional spectrophotometer. Additionally, our samples were not substantively pre-processed; samples were simply excised from an exposed site on a mouse, slightly cleaned of hair or excess blood, and subsequently placed under the fluorescence device. Naturally, these conditions would lead to noisier data, however, they also reflect operating room conditions more closely. Following this logic, while baseline intensity differences between spectra are important for classification, as shown in Fig 5A, they can be confounded by ambient light conditions, changing objective lens distance to sample, and other variables, thus leading one to question if classification model performance is truly based on pure physiological indicators and ultimately translatable to clinical environments. Because of this, it is also important to look at post-normalization differences, as shown in Fig 5B, to ascertain whether specific metabolic markers are present at different levels in unprocessed sarcoma and healthy tissue, as specified by the Warburg Effect.

It is important to note how few epochs are necessary for optimal ANN training. Because this is a relatively simple binary classification task for an ANN, Fig 9B shows optimal training around 35 epochs, with validation loss values substantially increasing beyond this epoch number, and Fig 9A showing gradually worse and more variable validation accuracy values beyond this epoch number. This finding demonstrates the potential misuse of neural networks in these settings, as they are computationally intensive and unnecessary in comparison to more readily interpretable and point-of-care models like SVM and LR.

Our device and evaluation pipeline outline the first known method, to our knowledge, for rapid sarcoma assessment using near-ultraviolet autofluorescence and interpretable machine learning, showing the potential translation of such a pipeline into clinics to rapidly provide oncosurgeons with automated non-contact validation while they work to delineate malignant tissue in a constantly changing exposure site.

Though machine learning models were chosen with specific attention given to their applicability for this specific classification problem, and each architecture was adequately cross validated and optimized with respect to hyperparameters, there is room for further improvement of classification algorithms. Support Vector Machines and Logistic Regression are known methods for dealing with heavily overlapping or multidimensional data, such as this. Support Vector Machines build a hyperplane in a higher dimension to draw differences not always evident in a standard three dimensions, and Logistic Regression converts class probabilities through a logits operator into binary classifications. However, other methods such as Random Forest and Gradient Boosting are possible, though they are slightly less interpretable than SVM or Logistic Regression, so it is difficult to tell if classifications are based on verifiable physiological differences, as demonstrated by SVM and LR. These former algorithms also perform worse than neural network frameworks for this particular problem; due to these issues, these two model choices were left out of the study. Though convolutional neural networks could have been utilized to achieve even better performance than an ANN, they still lack the ability to be interpreted, and LR and SVM were shown to perform similarly enough to the neural network structure for this study, thus diminishing the need to implement convolutional neural networks.

This device and classification pipeline should next be brought into operating rooms for collection on freshly resected human cancer tissue to validate this method as a potential tool for surgeons and streamline surgeries. It can also be compared with surgeon performance to investigate the device and algorithm’s potential as an assistive tool for surgeons as they operate, or as a tool to streamline pathology testing. The automated nature of supervised learning methods in this study show the potential for pairing with other devices, such as imaging modalities and actuational devices, to provide a closed loop system for tumor removal at ambiguous boundaries.
